# Endemisation and management of *Babesia divergens* on a beef production farm

**DOI:** 10.1016/j.crpvbd.2024.100188

**Published:** 2024-06-14

**Authors:** Andrea Springer, Daniela Jordan, Martin Höltershinken, Dieter Barutzki, Christina Strube

**Affiliations:** a Institute for Parasitology, Centre for Infection Medicine, University of Veterinary Medicine Hannover, Buenteweg 17, 30559, Hanover, Germany; b Clinic for Cattle, University of Veterinary Medicine Hannover, Bischofsholer Damm 15, 30173, Hanover, Germany; c Tierärztliches Labor Freiburg GmbH, Engesserstraße 4b, 79108, Freiburg, Germany

**Keywords:** Bovine babesiosis, Piroplasmosis, Tick-borne diseases, *Ixodes ricinus*

## Abstract

The hard tick *Ixodes ricinus* transmits a variety of zoonotic pathogens, including *Babesia divergens*, the most common cause of bovine babesiosis in northern Europe. In endemic areas, cattle are rarely clinically affected, as animals up to the age of nine months are resistant against relevant clinical disease and develop protective premunity. However, outbreaks in immunologically naïve herds may lead to considerable losses. Such an outbreak with a high mortality rate occurred in 2018 on a northern German beef production farm, as previously reported. The present study provides an update on the epidemiological situation and management strategy of the farm. In spring 2022, blood samples were taken from 46 animals for PCR and serological testing before pasture turnout. Although no clinical cases had been noticed since 2019, *B. divergens* DNA was detected by quantitative real-time PCR (qPCR), followed by amplification and sequencing of the 18S rRNA gene, in 6.5% (3/46) of cattle blood samples. Presence of anti-*B. divergens* antibodies was confirmed in 26.1% (12/46) of animals, while further 10.9% (5/46) had a borderline antibody titre. The antibody status of 23 of these animals had already been determined in 2018 and/or 2020, revealing fluctuating titre patterns indicative of repeated pathogen exposure. Moreover, 457 questing *I. ricinus* specimens collected on the farm’s pastures and 83 *I. ricinus* specimens detached from cattle were screened for *Babesia* spp. DNA by qPCR, followed by 18S rDNA amplification and sequencing. Endemisation of *B. divergens* was confirmed by 0.9% (4/457) positive questing *I. ricinus*, while the ticks detached from cattle were *Babesia*-negative. The farm’s management strategy includes annual metaphylactic treatment with imidocarb dipropionate during the main tick exposure period in spring. However, the antibody titre fluctuations and the persistent infections at the end of the housing period indicate that the absence of clinical disease is primarily due to a rising level of premunity. Metaphylactic treatment with imidocarb seems to be a suitable management option to protect newly acquired immunologically naïve animals. The endemisation of *B. divergens* is also of public health significance, as the pastures are located close to a tourist destination in a popular hiking area.

## Highlights

• Bovine babesiosis due to *Babesia divergens* initially affected a German farm in 2018.
• Four years later, persistent infections were detected at the end of the housing period.
• Fluctuating antibody titres indicated repeated pathogen exposure.
• Endemisation of *B. divergens* was confirmed by 0.9% positive questing *Ixodes ricinus.*
• Results are of public health significance due to the zoonotic potential of *B. divergens.*

## Graphical abstract

**Figure:**
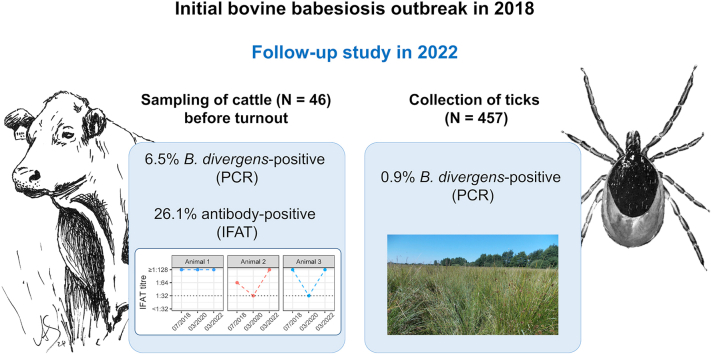

## 1 Introduction

In Europe, the widespread hard tick *Ixodes ricinus* acts as the principal vector of zoonotic tick-borne pathogens. Among them, the piroplasmid parasite *Babesia divergens* primarily infects cattle, but also represents the most common cause of human babesiosis in Europe (Hildebrandt et al., 2021). In susceptible cattle, infection leads to potentially fatal haemolytic anaemia with elevated body temperature, weakness, tachycardia, tachypnoea and haemoglobinuria (Sherlock et al., 2000; Zintl et al., 2003). However, animals up to the age of approximately nine months experience only mild or asymptomatic infections, but develop protective immunity, which is maintained by repeated pathogen exposure (Gern et al., 1988; Zintl et al., 2005). Therefore, clinical cases rarely occur in endemic regions, mainly affecting immunologically naïve animals, e.g. those acquired from other areas (Zintl et al., 2003). Human cases, although rather rare, have been reported involving immunocompromised/splenectomised as well as immunocompetent individuals, with the most severe symptoms and a high mortality rate in splenectomised patients (summarized by Hildebrandt et al., 2021).

Once of considerable economic impact in the livestock sector, the prevalence of *B. divergens* in cattle seems to have decreased during the last decades in several countries and clinical cases of bovine babesiosis are rarely reported (Hornok et al., 2006; Hasle et al., 2010; Zintl et al., 2014). However, a rather high incidence was determined during an enhanced surveillance effort in the UK in 2021 (McFadzean et al., 2023), indicating that the low number of cases in other countries, where no recent investigations on *B. divergens* epidemiology were performed, might also be due to underreporting. Furthermore, recent studies have reported an altered seasonal pattern, with unusual occurrence of outbreaks during the winter months (Johnson et al., 2020), as well as increased disease severity and case fatality, indicating a loss of endemic stability and possibly a lower awareness for the disease (Zintl et al., 2014; Springer et al., 2020).

Such an outbreak with a high mortality rate occurred in 2018 on a farm in northern Germany with no previous history of bovine babesiosis (Springer et al., 2020). Initially, one of five extensively managed suckler herds was affected, with a total loss of 21 animals due to a delay in diagnosis and treatment. Despite this large number of affected animals, PCR-based screening of 1430 ticks collected during 2018 and 2019 from the affected pasture and surrounding areas did not result in detection of *B. divergens*. Nevertheless, serological investigations in March 2020 revealed anti-*B. divergens* antibodies in 37.9% of the farm’s stock (Springer et al., 2020). The present study provides an update on the epidemiological situation on the farm four years after the initial outbreak, including molecular screening of cattle as well as ticks for *B. divergens* infection, and determination of anti-*B. divergens* antibody titres in cattle. Moreover, the farm’s management strategy is discussed.

## 2 Materials and methods

### 2.1 Farm characteristics and outbreak history

In 2018, an organic beef production farm in northern Germany was affected by an outbreak of bovine babesiosis as reported previously (Springer et al., 2020). During the summer period, the farm employs an extensive grazing system based on natural pastures located near a nature reserve, while the permanent stock is kept in loose housing during the winter months. Each spring, new herds, i.e. grazing groups, are formed and turned out to pasture. Briefly, the initial outbreak in May–June 2018 affected one of five suckler herds, resulting in the death of 21 cattle (Springer et al., 2020). Further clinical cases occurred on two other pastures during 2019, suggesting a geographical spread of the pathogen on the farm. Consequently, metaphylactic imidocarb dipropionate treatment of cattle was initiated in 2019. Animals over the age of nine months turned out to previously affected pastures receive a single dose (0.85 mg/kg, Imizol®, Intervet UK Ltd., Milton Keynes, UK) during the main activity season of *I. ricinus* in spring.

### 2.2 Blood sampling and processing

At the end of the winter housing period and before pasture turnout in March 2022, blood samples were taken from 46 mostly adult (> 3 years-old) cattle for diagnostic purposes at the request of the animal owner. The samples were collected from the jugular vein into serum and EDTA tubes and stored at 8 °C until further processing. Blood smears were prepared from EDTA blood, air-dried, fixed with methanol and stained with Giemsa solution before microscopic examination. Serum was obtained by centrifugation at 2000×*g* for 10 min and frozen at −20 °C until serological testing with a commercial indirect immunofluorescence test (MegaFLUO® *Babesia divergens*, MEGACOR Diagnostik GmbH, Hoerbranz, Austria). According to the manufacturer’s instructions, titres ≥ 1:64 were considered positive, while a titre of 1:32 was considered borderline and a titre of < 1:32 was considered negative.

The remaining blood clots were subjected to DNA isolation using the NucleoSpin 8 Blood kit (Macherey-Nagel GmbH & Co. KG, Düren, Germany) according to the manufacturer’s instructions.

### 2.3 Tick collection and nucleic acid extraction

**Fig. 1:**
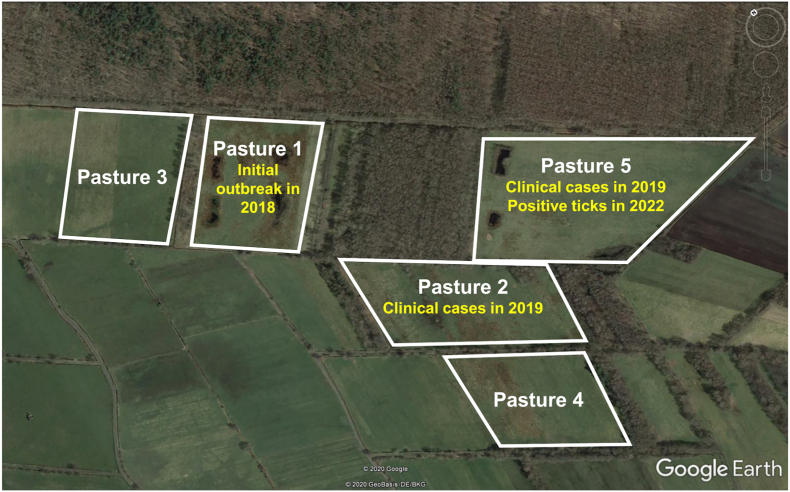
Satellite image of the pastures where clinical bovine babesiosis cases occurred in 2018/2019 and *B. divergens*-positive ticks were detected in 2022. Figure modified from Springer et al. (2020).

Questing ticks were collected by the flagging method during May and October 2022 on the pasture where the last clinical cases had occurred during 2019 (Pasture 5, Fig. 1). Additionally, attached ticks were collected from the farm’s cattle on different pastures while they were restrained for imidocarb treatment in May 2022. After microscopic identification according to published keys (Estrada-Peña et al., 2017), individual adult and nymphal ticks as well as pools of up to ten larvae were subjected to DNA isolation using the NucleoSpin 8 Blood kit (Macherey-Nagel GmbH & Co. KG, Düren, Germany) as described previously (Tappe and Strube, 2013). Genomic DNA was stored at −20 °C until further use.

### 2.4 *Babesia* screening by genus-specific qPCR and conventional PCR

Screening of blood samples and ticks for *Babesia* spp. (excluding *B. microti* and *B. microti*-like) was performed by SYBR Green-based qPCR targeting the mitochondrial lsu5-lsu4 region with primers B-Isu-F and B-Isu-R2 (Qurollo et al., 2017) (Table 1). The 25 μl reaction included 5 μl (blood samples) respectively 10 μl (ticks) DNA template, 12.5 μl iTaq™ Universal SYBR® Green Supermix (Bio-Rad Laboratories GmbH, Feldkirchen, Germany) and 0.5 μl of each primer (30 μM each). The thermoprofile consisted of 95 °C for 10 min, followed by 40 cycles of 95 °C for 15 s and 60 °C for 1 min, with subsequent melting temperature (T_m_) measurements between 55 and 95 °C. All qPCRs included a negative control and plasmid DNA containing the targeted *B. divergens* lsu5-lsu4 region, obtained from a *B. divergens*-positive clinical sample, as a positive control.

**Table 1:** Molecular assays used for *Babesia* spp. detection and differentiation in cattle blood samples and *Ixodes ricinus* ticks from a farm in northern Germany.

Assay type	Target sequence	Primers	Reference
SYBR Green-based qPCR	Mitochondrial lsu5-lsu4 region	B-lsu-F: 5′-ACCTGTCAARTTCCTTCACTAAMTT-3′	Qurollo et al. (2017)
		B-lsu-R2: 5′-TCTTAACCCAACTCACGTACCA-3′	
Semi-nested conventional PCR	18S rDNA	BJ1: 5′-GTCTTGTAATTGGAATGATGG-3′	Casati et al. (2006)
		BN2: 5′-TAGTTTATGGTTAGGACTACG-3′	
		PiroB: 5′-TTAAATACGAATGCCCCCAAC-3′	Armstrong et al. (1998)

To confirm positive qPCR results and to achieve *Babesia* spp. differentiation, positive samples with a T_m_ of 73.1–78.1 °C were subjected to a semi-nested conventional PCR targeting a ∼350 bp fragment of the 18S rRNA gene with primers BJ1 and BN2 (Casati et al., 2006) in the first round and BJ1 and PiroB (Armstrong et al., 1998) in the second round (Table 1). The 25 μl reaction of the first round contained 5 μl of DNA template, 2.5 μl 10× buffer, 0.5 μl of dNTPs (10 mM each), 0.5 μl of each primer (10 μM each) and 0.25 μl DreamTaq® polymerase (5 U/μl, Fisher Scientific GmbH, Schwerte, Germany). In the second round, 1 μl of the PCR product from the first PCR was used as template. The thermoprofile for both rounds consisted of 95 °C for 3 min, 40 cycles of 94 °C for 30 s, 55 °C for 30 s, 72 °C for 1 min and final elongation at 72 °C for 10 min.

Amplicons were visualized by electrophoresis on 1.5% agarose gels stained with GelRed (Biotium Inc., Fremont, CA, USA), custom Sanger-sequenced (Microsynth Seqlab Sequencing Laboratories, Göttingen, Germany) and compared to publicly available sequences using NCBI BLAST.

## 3 Results

### 3.1 *Babesia* infection status and antibody titres of housed cattle in March 2022

Of the 46 cattle sampled before turnout in March 2022, i.e. after an indoor period of at least four months, seven were positive for *Babesia* spp. by SYBR Green-based qPCR. For three (6.5%) of these animals, 18S rDNA sequences could be generated (GenBank accession nos: PP759293-PP759295), which were 100% identical to *B. divergens* (including the chosen reference sequence U16370; 100% query cover [QC]). In contrast, no *B. divergens* stages were noted in the blood smears of any of the animals.

Presence of anti-*B. divergens* antibodies was confirmed in 26.1% (12/46) of animals, while further 10.9% (5/46) had a borderline antibody titre of 1:32 and 63.0% were seronegative (29/46). Interestingly, all three animals with successful *B. divergens* sequencing results were seronegative. Of the remaining qPCR-positive animals, two were seropositive with a titre of 1:512 and 1:256, respectively, one had a borderline titre and one was seronegative.

**Fig. 2:**
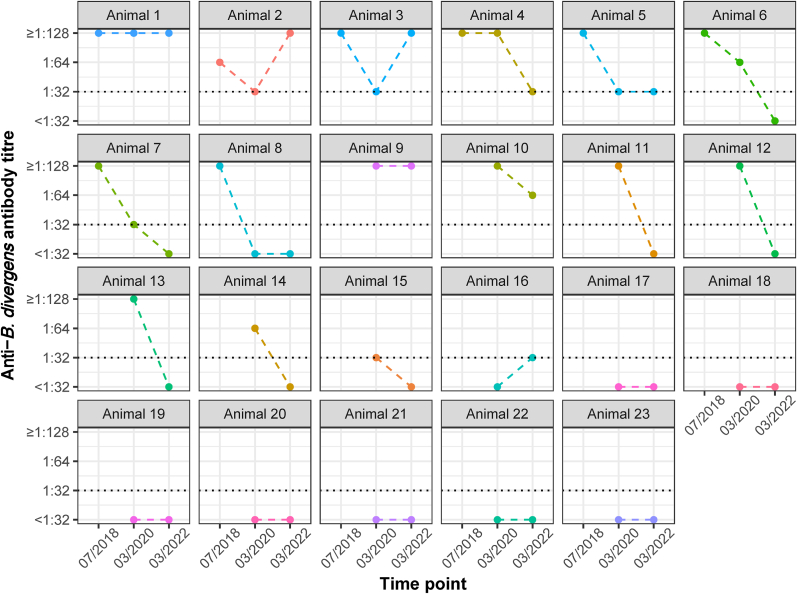
Anti-*B. divergens* antibody titres in repeatedly tested cattle from a beef production farm in northern Germany. The dashed line indicates a borderline titre of 1:32.

The antibody status of 23 of the 46 tested animals had already been determined in July 2018 and/or March 2020 (Springer et al., 2020). Five (21.7%) animals had seropositive titres at earlier samplings and again in 2022 (Fig. 2). Two of these showed a fluctuating titre with a decrease from 2018 to 2020 and a subsequent increase. In ten (43.5%) animals, a titre decline from a seropositive or borderline to a borderline or seronegative result in 2022 was observed, while seven (30.4%) remained seronegative, and one animal (4.3%) showed a titre increase from a seronegative to a borderline result (Fig. 2).

### 3.2 *Babesia divergens* detection in ticks

In total, 428 individual questing *I. ricinus* (350 nymphs, 29 females and 49 males) and three pools containing a total of 29 larvae were subjected to *Babesia* screening by qPCR (Table 2). DNA of *B. divergens* was confirmed in four specimens: two adult males and one adult female collected in May 2022 and one nymph collected in October 2022. The obtained 18S rRNA gene (GenBank accession nos: PP759296-PP759299) sequences showed 99.7–100% identity to published *B. divergens* sequences in GenBank (including the chosen reference sequence U16370, 100% QC). Other *Babesia* spp. were not detected.

**Table 2:** Prevalence of *Babesia divergens* in questing *Ixodes ricinus* ticks collected on a farm affected by bovine babesiosis.

Month of sampling	Developmental stage	*B. divergens*-positive ticks/tested ticks	Prevalence (%)
May 2022	Adult females	1/22	4.5
May 2022	Adult males	2/25	8.0
May 2022	Nymphs	0/233	0
May 2022	Larvae	0/29^a^	0
Total May 2022		3/309	1.0
October 2022	Adult females	0/7	0
October 2022	Adult males	0/24	0
October 2022	Nymphs	1/117	0.9
Total October 2022		1/148	0.7
Total both seasons		4/457	0.9

a Larvae were tested in three pools containing 9–10 specimens.

Moreover, 83 ticks (69 females, six males and eight nymphs) in various stages of engorgement were collected from a total of 34 cattle in May 2022. Of these, 41 ticks (36 females, three males, two nymphs) from 18 cattle were collected on Pasture 5, where the *B. divergens*-positive questing ticks were found (Fig. 1). The remaining 42 ticks (33 females, three males, six nymphs) were collected from 16 cattle on more distantly located pastures. None of the host-attached ticks were positive for *Babesia* spp.

## 4 Discussion

The present study investigated *B. divergens* infections in the cattle and tick host on a farm that was affected by an initial bovine babesiosis outbreak four years earlier. While this initial outbreak in 2018 was characterized by a high mortality rate, followed by sporadic clinical cases in 2019 (Springer et al., 2020), no morbidity was noticed on the farm during 2020 and 2021. As every year, the permanent stock was housed during the winter months of 2021/2022. Blood samples were taken before turnout in March 2022 and subjected to *Babesia* spp. PCR and serological testing. For three animals, *B. divergens* infections were confirmed by 18S rDNA sequencing, while four further animals were qPCR-positive, but no 18S rDNA sequence could be generated. Aside from *B. divergens*, *Babesia major* is the only other bovine *Babesia* species which occurs in Germany; however, its presence has only ever been described on certain islands in the North Sea (Liebisch et al., 1976). Its vector *Haemaphysalis punctata* is extremely rare in Germany, with only two sporadic reports from the mainland in addition to the populations described on the mentioned islands (Rubel et al., 2023). As cross-reactivity of the utilized qPCR assay with bovine DNA has not been observed (Qurollo et al., 2017), it is highly likely that all qPCR-positive animals in the present study were infected with *B. divergens*. In the examined blood smears, no *Babesia* stages could be observed; however, low parasitaemia has been described previously for asymptomatic *B. divergens* infections (Malandrin et al., 2004). In experimental studies, persistent infections lasting for several years after a single inoculation have been demonstrated (Joyner and Davies, 1967). Therefore, it was not surprising that animals were still infected after the four-month housing period, although new infections, e.g. iatrogenic by sharing of needles, cannot be ruled out. Nevertheless, a similar observation of persistent infections at the end of the winter period, nine months after acute infections, was made by Malandrin et al. (2004).

Antibodies against *B. divergens* may persist for at least one year even without reinfections (Christensson and Morén, 1987). However, in previous field studies, a decline in titres over the winter period has been repeatedly observed (LʼHostis et al., 1997; Malandrin et al., 2004). In the present study, 37.0% of tested animals still had positive or borderline antibody titres at the end of the winter period. It was not known whether all tested animals had grazed on affected pastures during the past two years, therefore, some of the seronegative animals may not have been exposed. The antibody status of 23 animals had already been determined two years earlier, in March 2020, and eight of them had additionally been tested in 2018, shortly after the initial outbreak. While most animals showed a similar or lower titre in 2022 than when tested previously, two of them were seropositive in 2018, borderline in 2020 and seropositive again in 2022. These titre fluctuations likely indicate repeated exposure during the study period, and variations in the antigenic stimulus between individual years. While reactivation of persistent infections may in theory also have caused the titre rise, this phenomenon has not yet been described for *B. divergens* in cattle to the authors’ knowledge.

Interestingly, some of the animals with molecularly confirmed infections were considered antibody-negative in serological testing. Although most subclinically infected animals were also seropositive in a previous study, absence of antibodies despite a positive *B. divergens* culture result has been occasionally observed (Malandrin et al., 2004). As little experimental research has been performed on *B. divergens* during the past 20 years, the relationship between antibody titres, presence of parasites and the degree of protection is still not clear, as stated by Zintl et al. (2003).

While previous efforts to confirm the occurrence of *B. divergens* in *I. ricinus* ticks from various pastures of the farm during 2018 and 2019 were not successful (Springer et al., 2020), 0.9% of 459 questing ticks were found to be infected in the present study, precisely three adult ticks in May 2022 and one nymph in October 2022. Although a lower number of ticks was collected in October compared to May, especially regarding adult females, the seasonal detection frequency was almost equal with 1.0% and 0.7%, respectively. A similar prevalence of 1.0% *B. divergens*-infected *I. ricinus* nymphs was determined in a recent study from Ireland, a country particularly affected by bovine babesiosis (McKiernan et al., 2022). This confirms the endemisation of *B. divergens* in the farm’s tick population and may indicate an increase in prevalence between the two surveys, which might be expected due to transovarial transmission in ticks (Joyner et al., 1963; Donnelly and Peirce, 1975). However, the results of the two studies are not completely comparable due to methodological differences. In 2018/2019, an equal *Babesia* spp. prevalence of 0.9% was detected among 1430 collected ticks, but only *B. microti*, *B. venatorum* and *B. capreoli* were identified. This first survey relied on a conventional PCR (Springer et al., 2020), while a SYBR Green-based qPCR was performed for screening in the present study. The target of the employed qPCR is located in the mitochondrial genome, which improves sensitivity compared to other assays (Qurollo et al., 2017). However, as the study primarily targeted *B. divergens*, only primers amplifying members of the *Babesia* sensu stricto and the so-called Western clade were included (Qurollo et al., 2017), while specific primers for *B. microti* and *B. microti*-like organisms were not contained in the reaction setup, precluding their detection in the present study. Following qPCR, positive samples were confirmed by amplification and sequencing of the 18S rDNA, as false positives due to cross-reactions with tick DNA have been observed (this issue: Springer et al., 2024). In most cases, a semi-nested protocol was necessary to generate a visible amplicon. Therefore, *B. divergens* infections may have been missed in the previous survey due to a less sensitive, non-nested PCR assay.

In other domestic animal species, such as dogs, prevention of babesiosis focuses on tick prophylaxis, with a multitude of available acaricides including long-acting products (Solano-Gallego et al., 2016). In contrast, only a flumethrin pour-on formulation is licensed against ticks in cattle in Germany, which needs to be applied every three weeks to achieve continuous protection. The extensive management system of the affected farm precludes such a high treatment frequency, wherefore no tick prophylaxis is currently in place. Likewise, avoidance of the affected pastures is not feasible. Therefore, management advice given to the farmer included a turnout of calves under nine months of age to previously affected pastures to increase the level of premunity among the stock. The owner additionally opted for annual metaphylactic imidocarb dipropionate treatment of adult animals on affected pastures during the peak tick season. This may reduce the risk for development of clinical babesiosis but does not prevent exposure, as demonstrated by the persistent infections detected at the end of the housing period in 2022 and by the fluctuating antibody titres. The necessity of these blanket treatments seems questionable, entailing rather high costs, a long withdrawal period and the risk of resistance development. Moreover, adverse effects such as muscular tremor, hypersalivation and colic are possible (MSD Animal Health, 2021). Annual antibody testing was suggested as an alternative strategy to the animal owner but may be similarly costly as only labour-intensive IFAT testing is available. In this light, annual PCR testing may be considered as well. Samples should preferably be taken at the end of the pasture season in late autumn. In the following spring, prophylactic treatment could then be limited to PCR- or antibody-negative animals, respectively, in addition to newly acquired animals.

If available, vaccination would be a preferred management option. Live-attenuated vaccines were available in some European countries in the past, but their production has been discontinued (Florin-Christensen et al., 2014). Several laboratories were contacted in the present case to ask whether production of a herd-specific vaccine would be possible, but no positive response was received.

The endemisation of *B. divergens* in the studied area does not only present a challenge for the farmer but is also of public health concern due to the zoonotic relevance of *B. divergens*. Although human babesiosis cases are rare in Europe, they often present as severe, life-threatening infections, particularly in splenectomised or otherwise immunocompromised patients (Hildebrandt et al., 2021). The pastures border a nature reserve, which is a popular tourist destination with many hiking trails. Apart from cattle, red deer have been suggested as potential reservoir hosts (McKiernan et al., 2022), which might be responsible for spreading the pathogen within the area, in addition to translocation of infected ticks by various wildlife.

## 5 Conclusions

Four years after the initial outbreak of bovine babesiosis, endemisation of *B. divergens* was confirmed in the farm’s *I. ricinus* population. The antibody titre fluctuations and the persistent infections in cattle at the end of the housing period indicate that the absence of clinical disease is primarily due to a rising level of premunity. Metaphylactic treatment with imidocarb seems to be a suitable management option to protect newly acquired immunologically naïve animals. Considering *B. divergens* epidemiology, the farm may represent a source of further pathogen spread by translocation of infected ticks or infected cattle, which is of concern both from a veterinary and from a public health perspective.

## Funding

The study was supported by a grant of the 10.13039/501100000780European Union through the European Regional Development Fund and the 10.13039/100013276Interreg North Sea Region Programme 2014–2020 as part of the NorthTick project (reference number J-No.: 38-2-7-19).

## Ethical approval

Ethical review and approval were not required because all samples from animals in the present study were taken for diagnostic purposes for the benefit of the animals and at the request of the animal owner. Informed consent was obtained from the owner.

## CRediT authorship contribution statement

**Andrea Springer:** Investigation, Visualization, Writing – original draft. **Daniela Jordan:** Investigation, Writing – review & editing. **Martin Höltershinken:** Investigation, Writing – review & editing. **Dieter Barutzki:** Investigation, Writing – review & editing. **Christina Strube:** Conceptualization, Supervision, Writing – review & editing.

## Declaration of competing interests

The authors declare the following financial interests/personal relationships which may be considered as potential competing interests: Dieter Barutzki acted as Managing Director of the diagnostic Tierärztliches Labor Freiburg GmbH and had research collaborations with veterinary pharmaceutical companies; Christina Strube has repeatedly lectured for and acted as a consultant for diagnostic and (veterinary) pharmaceutical companies and has previous and ongoing research collaborations with various diagnostic and (veterinary) pharmaceutical companies. The other authors declare that they have no known competing financial interests or personal relationships that could have appeared to influence the work reported in this paper.

## Data Availability

The data supporting the conclusions of this article are included within the article. Sequences generated during this study have been submitted to NCBI GenBank database under the accession numbers PP759293-PP759299.
